# Optimization of quasi-hemispherical CdZnTe detectors by means of first principles simulation

**DOI:** 10.1038/s41598-023-30181-2

**Published:** 2023-02-24

**Authors:** Valentina Vicini, Silvia Zanettini, Nicola Sarzi Amadè, Roman Grill, Nicola Zambelli, Davide Calestani, Andrea Zappettini, Leonardo Abbene, Manuele Bettelli

**Affiliations:** 1 IMEM-CNR, Parco Area Delle Scienze 37A, 43124 Parma, Italy; 2 Due2Lab S.R.L, Via P. Borsellino 2, 42019 Scandiano, RE Italy; 3 Institute of Physics, Faculty of Mathematics and Physics, Charles University, Ke Karlovu 5, 12116 Prague 2, Czech Republic; 4 Department of Physics and Chemistry (DiFC)-Emilio Segrè, University of Palermo, 90128 Palermo, Italy

**Keywords:** Computational methods, Materials for devices

## Abstract

In this paper we present the development of quasi-hemispherical gamma-ray detectors based on CdZnTe. Among the possible single-polarity electrode configurations, such as coplanar, pixelated, or virtual Frisch-grid geometries, quasi-hemispherical detectors are the most cost-effective alternative with comparable raw energy resolution in the high and low energy range. The optimal configuration of the sensor in terms of dimension of the crystals and electrode specifications has been first determined by simulations, and successively validated with experimental measures. Spectra from different sources have been acquired to evaluate the detectors performances. Three types of detectors with different CZT volumes have been fabricated, namely 10 × 10 × 5 mm^3^, 15 × 15 × 10 mm^3^ and 20 × 20 × 10 mm^3^. In the case of 10 × 10 × 5 mm^3^ crystals, the optimum pixel size determined by our simulation tool was confirmed by experiments: the best spectroscopic resolution of 1.3% at 662 keV has been found for a 750 μm diameter pixel detector. The best energy resolution values obtained for the 15 × 15 × 10 mm^3^ and 20 × 20 × 10 mm^3^ detectors were respectively 1.7% and 2.7% at 662 keV.

## Introduction

The high atomic number, high density, broad band gap, low chemical reactivity, and long-term stability of Cadmium Zinc Telluride (CdZnTe or CZT) make it an outstanding material choice for high efficiency, high-resolution room-temperature nuclear radiation detectors. In the last decades, there has been a lot of work done on CdZnTe crystal growth and electrical compensation, which led to the fabrication of CdZnTe crystals with low defect density, that translates into better charge transport capabilities, in particular for electrons (mobility lifetime product greater than 10^–2^ cm^2^/V^1^). CdZnTe is now commercially available from several sellers and its characteristics allows it to be used in a wide range of applications: medical, industrial, security, safeguarding, and scientific applications.

The capability of CdZnTe detectors to operate at room temperature makes them very appealing in applications where lightweight and compact devices are needed^2,3^. Recently, the scientific community has demonstrated the possibility to grow large volume CdZnTe crystals (24 cm^3^) with high performances and high material homogeneity^4^. The use of bulky detectors is desirable in the sectors of nuclear safety and security applications^3,5^, where fine sensitivity is necessary rather than spatial resolution. In these applications, the newly developed detectors must be cost-competitive with typical nowadays used scintillators and the entire system must be compact and lightweight. This is why the opportunity to exploit large volume detectors with a small number of readout channels is particularly interesting: the aim is to keep the cost of electronics and integration, which often greatly affects the price of the final device, as low as possible.

In the years, several detector geometries have been studied in order to overcome the poor hole transport properties in CdZnTe, which could lead to inefficient and low performance devices. Pixelated detectors^6^ and orthogonal stripes detectors^7,8^ allow to neglect the hole contribution to the signal thanks to the “small pixel effect”, but they are unsuitable in the aforementioned applications due to the expensive readout tools. Coplanar^9^ and Virtual Frisch-Grid (VFG)^10^ detectors allow to exploit large volumes of CdZnTe with few electronic channels. However, both geometries are characterised by large parasitic capacitance.

Among all the electrode geometries, the quasi-hemispherical^11–14^ configuration allows to collect charges over large detector volumes (up to 4 cm^3^) with a single readout channel. Quasi Hemispherical Detectors (QHDs) do not need Pulse Shaping Analysis algorithm, as opposed to coplanar and VFG, and their state-of-the-art performances are achieved by using the raw spectrum only. Furthermore, their fabrication procedure is not critical since all electrodes can be deposited in a single step after photolithography.

Pixel size is crucial in QHDs^15^ since it influences both electric and weighing fields. On one side, a too small pixel could lead to a weak electric field in large detector regions, which causes long drift times and, consequently, poor and inhomogeneous charge collection. On the other side, a large pixel decreases the “small pixel effect”, and this could lead to the presence of peak tailing due to hole trapping.

Bao et al.^15^ studied 10 × 10 × 5 mm^3^ QHDs and they simulated the spectral response for quasi-hemispherical CdZnTe with different pixel sizes. However, the space charge inside the detectors, which modifies both the spatial distribution of electric field and the charge collection, was not taken into account, and the simulations were not validated with experiments.

The aim of this work was to identify the best electrode configuration for QHDs of different dimensions. Three series of detectors were bought from Redlen Technologies: 10 × 10 × 5 mm^3^ (0.5 cm^3^, named ‘V5’), 15 × 15 × 10 mm^3^ (2.2 cm^3^, named ‘V22’) and 20 × 20 × 10 mm^3^ (4.0 cm^3^, named ‘V40’). Firstly, a set of simulations was performed for the 0.5 cm^3^ crystals by changing the pixel size. Thanks to simulations, a wide pixel dimension range and its related performances have been explored. After the identification of the best pixel size, three V5 detector pairs with different pixel sizes were realised in order to verify the accuracy of simulations. A detailed characterization of V5 detectors was performed aiming to compare the results obtained by simulation and real detectors. Subsequently, V22 and V40 detectors were modelled and then fabricated according to the results of the simulator. The spectroscopic performances of all these detectors have been measured and reported in the paper.

## Methods

### First principles simulations

In order to identify the best contact configuration, a first principles simulator^16,17^ developed at IMEM-CNR laboratory was used. This simulation tool combines a Monte Carlo simulator (GEANT4), a Finite Elements Method (FEM) calculator and a numerical computation software. It is capable of reproducing the radiation-semiconductor interaction, the weighting/electric fields and the carrier transport/signal induction, respectively. This simulator does not require preliminary calibration measurements, thus providing large benefits in terms of development efforts and costs. The tool allows predicting the best detector design in terms of dimension, contact geometry and operating conditions: this translates in a real performance improvement.

The system simulates the transport of charges generated by photon-matter interactions in the electric field of the CZT sensor. The charge motion induces a signal (which is a transient electrical current) to the collecting electrode. The software calculates trajectories of both carrier types by means of ODE (Ordinary Differential Equation) functions. Once motion equations are known, the software calculates the induced current for both electrons and holes according to Ramo–Shockley theorem. The total collected charge is calculated by integrating simulated current signals. The last step is the binning of the total charge values and the extrapolation of the spectrum. The best detector geometry was determined comparing spectra generated by several simulated devices.

In this work the Geant4 block of the simulator has not been used because the aim was to compare the performances of several electrode geometries and identify the best among them, and not to simulate the spectrum related to a specific radioactive source or a specific experiment.

**Figure 1:**
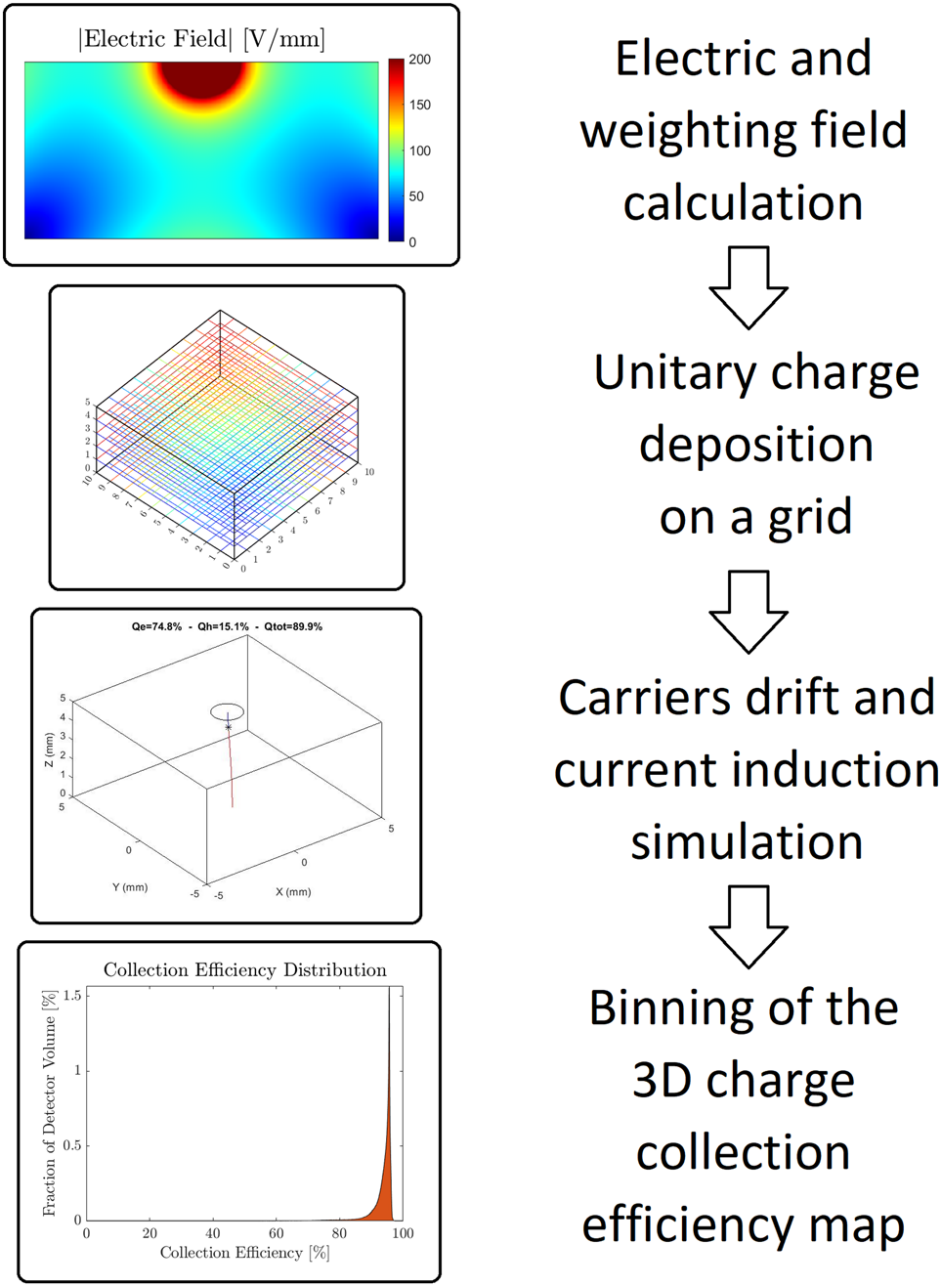
Schematization of the simulation process with a graphical representation of each of the four steps described in the text.

In order to simulate the detector performance, the following steps, outlined in Fig. 1, were carried out:

1. Electric and weighting fields were calculated (with the FEM calculator). In order to correctly model the electric field inside the detector, it was necessary to consider the effect of strong hole trapping that occurs in the vicinity of the anode. Laser-induced transient current measurements^18,19^ suggest that the anode space charge is about 10 nC/cm^3^ in steady state. Electric field was simulated by using COMSOL electrostatic package and by considering a space charge distribution obtained using the Space-Charge Limited Currents model^20^, solved as a classic Corona problem^21^. All simulations were performed thanks to COMSOL Multiphysics.
2. A uniform 3D grid was created inside the detector volume and a unitary positive and negative charge was deposited in each grid node. This strategy allowed to calculate the Charge Collection Efficiency (CCE) value for each node and generate the three-dimensional Charge Collection Efficiency Map (CCEM) which is fundamental to evaluate detector geometries avoiding statistical noise effects. Generally, the radiation-matter interactions inside the sensor volume are simulated with a Monte Carlo simulator based on GEANT4, but a different method is proposed in this work in order to compare the performance of different detectors.
3. Carriers drift and current induction were simulated and the CCEM was calculated. The charges generated in step (2) are drifted according to the simulated electric field, and the signal induced to the anode is computed by means of the Ramo–Shockley theorem^22^. The CCE value was calculated for each grid node after the integration of the induced signal, and this generated the 3D CCEM. This map allowed to identify dead or low efficiency regions in the detector volume.
4. The CCEM was evaluated by means of the Performance Array (PA), a histogram obtained by binning all the values of the CCEM. In an ideal case the obtained PA is a delta function centred in 1 (100% charge collection efficiency), while in a real case the carrier trapping causes a reduction of CCE, which is thus peaked below 100%.

The capability of the simulator to take into account the space charge naturally generated inside the detectors was crucial. It has been found that the transit time resulting from simulations without considering the presence of space charge was much longer than the real transit time experimentally observed by laser-induced transient current measurements (for example the electron transit time in a detector simulated in absence of space charge and biased at 150 V is 4.1 µs, while the corresponding measured transit time is approximately 0.7 µs). On the contrary, experimental and simulated values were in agreement when the electric field computed in step 1 considers the presence of space charge. The simulations performed in this paper have been carried out considering a boundary condition value for the anode space charge equal to 10 nC/cm^3^, deduced from laser-induced transient current experiments.

Once the PA was extracted for each geometry, the Peak Width Evaluation Function (PWEF) was calculated according to the following formula. The function takes into account the Full Width at Half Maximum (FWQM), the Full Width at Quarter Maximum (FWQM) and the Full Width at One-eight Maximum (FWOM) and allows to evaluate the peak width at three different heights, with the goal of better estimate the tailing effect.

$$PWEF=\sqrt{\frac{{\left(FWHM-min\left(FWHM\right) \right)}^{2}}{{\left(FWHM\right) }^{2}}+\frac{{\left(FWQM-min\left(FWQM\right) \right)}^{2}}{{\left(FWQM\right) }^{2}}+\frac{{\left(FWOM-min\left(FWOM\right) \right)}^{2}}{{\left(FWOM\right) }^{2}}}$$

PWEF minimization was the criterion used to judge which was the most performing electrode geometry.

The simulator allows the optimisation of the detector geometries regardless of the incoming photons energy. While the simulator has been used in other experiments^16,17^ to compare simulated and measured spectra, this comparison was not necessary in this work, because the aim was the optimization of the detector geometry.

### Detector fabrication

Sensors were realised starting from CdZnTe crystals grown by Travelling Heater Method by Redlen Technologies. CdZnTe crystals were reprocessed following the standard procedure developed at IMEM-CNR and contact layout was determined by the simulations.

**Figure 2:**
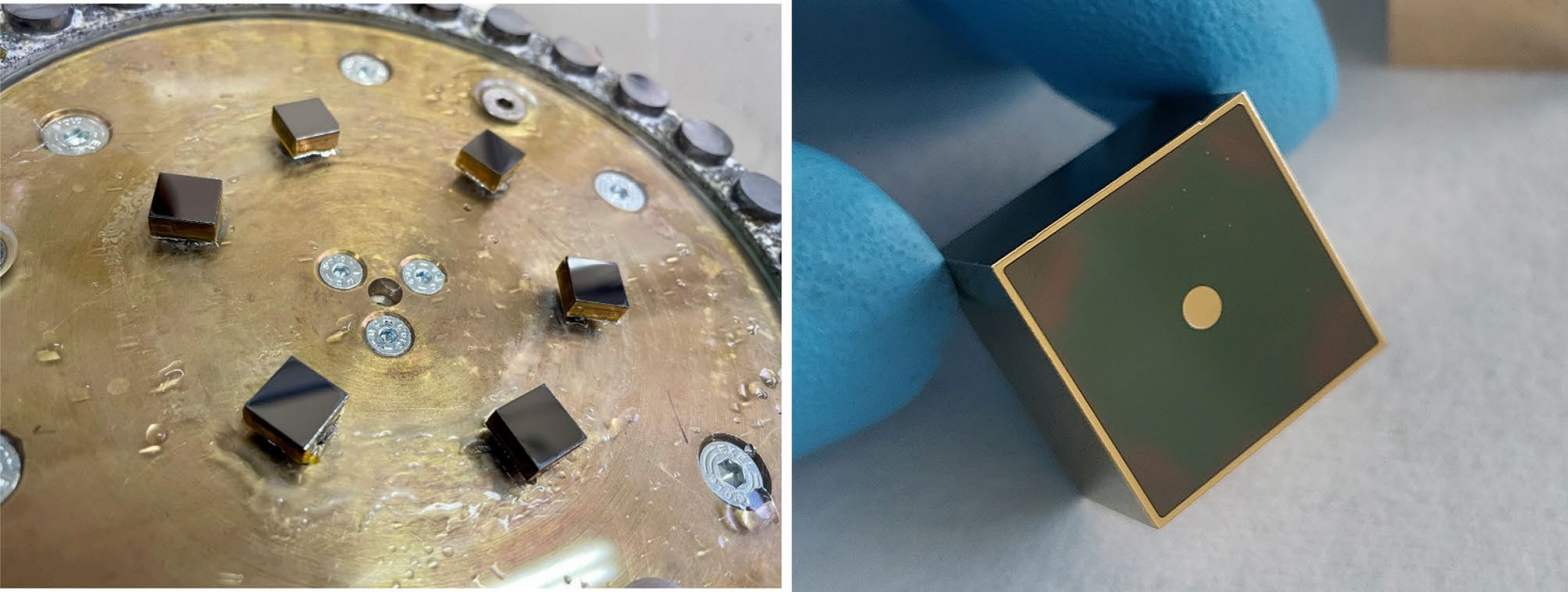
Six 10 × 10 × 5 mm^3^ detectors glued on the lapping jig (left); one of the 15 × 15 × 10 mm^3^ fabricated detectors (right).

Before contact deposition, surfaces were lapped by means of SiC-abrasive paper (grit size P1000 and P2500) and then chemo-polished (Fig. 2—left). Negative photoresist was used to realise the contacts patterning, obtained by means of anode surface photolithography. Au contacts were made by chemical electroless deposition from methanol solution^23^. In order to completely remove surface oxidation, a short Br-based etching was carried out right before Au deposition. This fabrication process characterised by photoresist patterning prior to gold electroless metallization has been already adopted for the fabrication of drift-stripes detectors^8,24^, for which it showed a reduction of surface leakage current by a factor 10 with respect to other passivation techniques.

Finally, the finalised detectors (Fig. 2—right) were inserted in a custom sample holder realised in ABS (Acrylonitrile Butadiene Styrene) with a 3D printer. Cathode and anode of the detector were wire-bonded on dedicated metallic pins installed on the sample holder. All the detectors were prepared using the same preparation steps.

### Electrical characterization

Dark leakage currents of detectors were measured before the spectroscopic performance evaluation, both before and after bonding. The measurements were carried out inside a dark Faraday cage to minimise the electric noise and photogenerated current, by means of soft-tips in contact with Au electrodes on CZT before bonding, and by using a dedicated socket with the detector assembly pinout once the sensors were bonded. Keithley sourcemeter 2410 was used as voltage source up to 1100 V, while a customised High-Voltage generator, able to reach 5000 V, was used to apply higher polarisations. The electric current was measured by means of a Keithley picoammeter 6548 that enables high precision measurements, down to the picoampere current range. Data were recorded on a computer connected with the instruments by using a PCIB-USB module.

### Spectroscopic characterization

In order to evaluate spectroscopic performances, the detectors were exposed to uncollimated gamma ray sources irradiating from the cathode side. Radioisotopes were chosen in order to cover a wide energy range, from a few tens of keV to more than 1 MeV. Tested sources were: Am-241, Ba-133, Co-57, Co-60, Cs-137, Na-22, U (10% U-235), Pu-239. During gamma-ray measurement, the detectors were biased by means of a customised High-Voltage generator, already used for current–voltage measurements. A digital readout electronics chain realised by Due2Lab s.r.l. was used to readout detector signals. The readout electronic chain is composed by a customised charge sensitive preamplifier (sensitivity 36 mV/MeV, about 100 electrons RMS, decay time: 20 µs), coupled with a fast ADC (100 Msample/s, 14 bit) and a FPGA unit, which converts the digitalized signals into the energy spectrum. No pulse shape analysis or correction was performed in order to better compare the real detectors performances.

## Results

### 10 × 10 ×  5 mm^3^ QHDs optimization

#### Simulations

The simulations have been restricted to quasi-hemispherical geometry only. Since the simulated detector dimensions were fixed in relation with the purchased crystal dimensions, the key parameter to be modified to evaluate its influence on the performance of the device is the pixel dimension. Seven pixel dimensions were simulated ranging from 100 μm to 2 mm of diameter.

**Figure 3:**
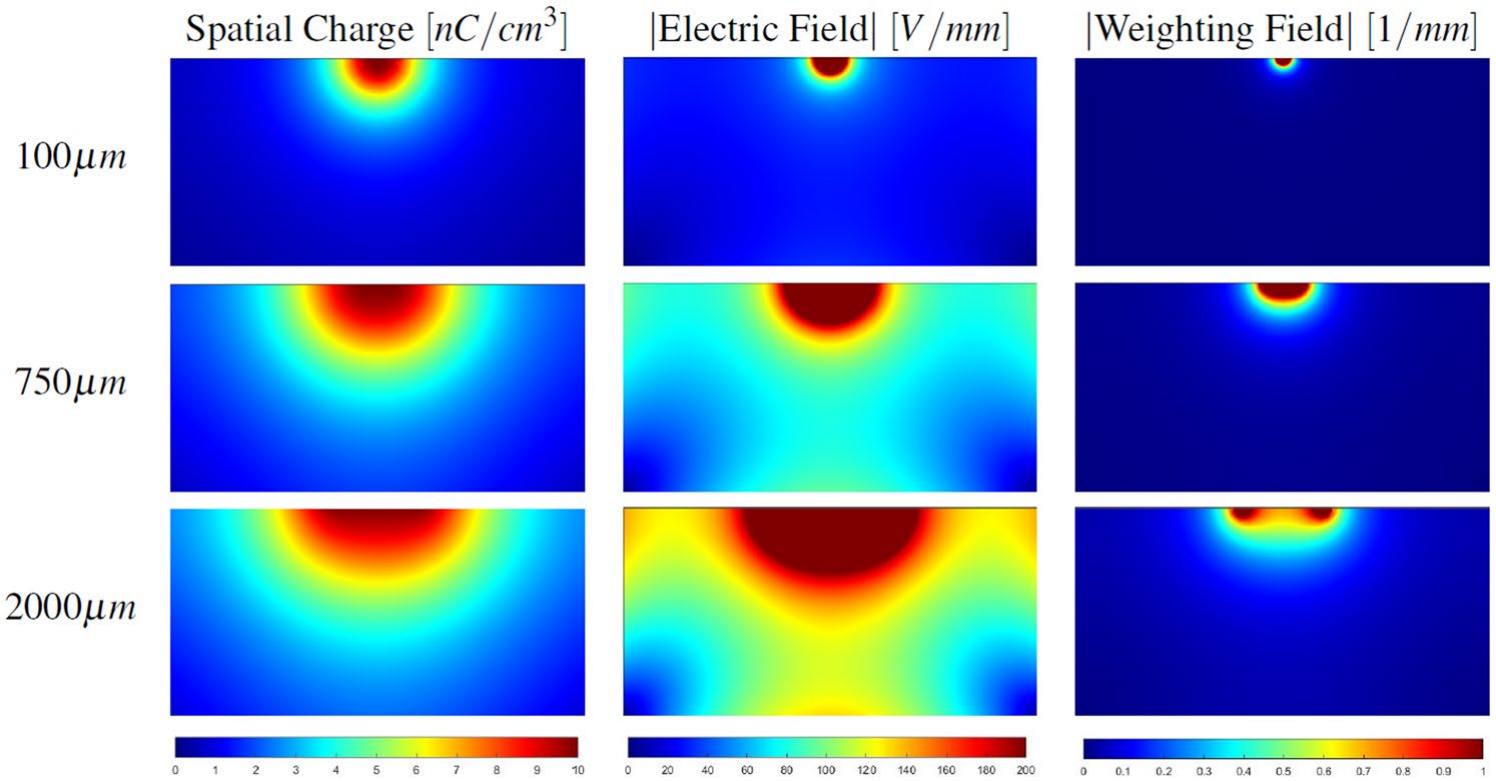
Space charge (left) Electric field (centre) and Weighting field (right) profiles evaluated on the YZ plane passing through the centre of the pixel are shown for 3 different pixel dimensions: 100 μm (top), 750 μm (centre), 2000 μm (bottom).

For each detector:

- Electric and weighting fields were computed by using COMSOL Multiphysics (Fig. 3), and their values were calculated on a non-uniform 3D grid composed by 301 × 301 × 151 nodes (more than 13 M points) and exported on a txt file. The 3D grid non-uniformity was characterised by a higher density of points in the region near the pixel and was adopted in order to increase the simulation accuracy. Anode was grounded, while cathode was biased to − 1000 V for all simulated detectors.
- The CCEM was evaluated using a similar non-uniform 3D grid. Since CZT crystals were purchased from Redlen Technologies, standard carrier transport properties of spectroscopic Redlen CZT reported in Table 1 were used in the simulations.
- Values of CCEM were interpolated with a homogenous 3D grid (more than 3 million of nodes), which ensures a spatially uniform evaluation of each detector region.
- These obtained values were binned in 4096 bins from 0 to 1; the resulting Performance Array (PA) was normalised by its integral and by the width of a single bin, and represents the capabilities of the entire detector to collect charges. PA of an ideal detector that collects all photogenerated charges with no losses would be a delta function centred in 1. Typically, PAs of simulated detectors present a peak shifted to lower values respect 1 and broadened due to inhomogeneity of the detector response.

**Table 1:** Carrier transport parameters for electrons and holes for spectroscopic CZT by Redlen^25^.

Transport parameters	
${\mu }_{e}$	$1000 \; {\mathrm{cm}}^{2}/(\mathrm{V}\cdot \mathrm{s})$
${\mu }_{h}$	$88 \; {\mathrm{cm}}^{2}/(\mathrm{V}\cdot \mathrm{s})$
${\tau }_{e}$	$11 \; \upmu \mathrm{s}$
${\tau }_{h}$	$1.2 \; \upmu \mathrm{s}$

As shown in Fig. 3, one could expect that smaller pixels improve performances thanks to a stronger small-pixel effect (visible in weighting field plots). However, a too small pixel produces a weaker electric field that determines larger charge losses.

Figure 3 also shows the importance of including the space charge in the simulations. In the case of a hemispherical detector, if the space charge distribution is neglected, the electric field map becomes qualitatively equivalent to the weighting field. It is evident that, in such case, the electric field would be very low in most of the detector volume, causing slow carrier drift and strong trapping of electrons. As shown in next paragraphs, this scenario is not verified by experimental data.

In order to quantitatively compare the PAs of different detectors and to identify the optimal pixel dimension, the Full Width at Half Maximum (FWHM), the Full Width at Quarter Maximum (FWQM), and the Full Width at One-eight Maximum (FWOM) of PA have been evaluated.

**Figure 4:**
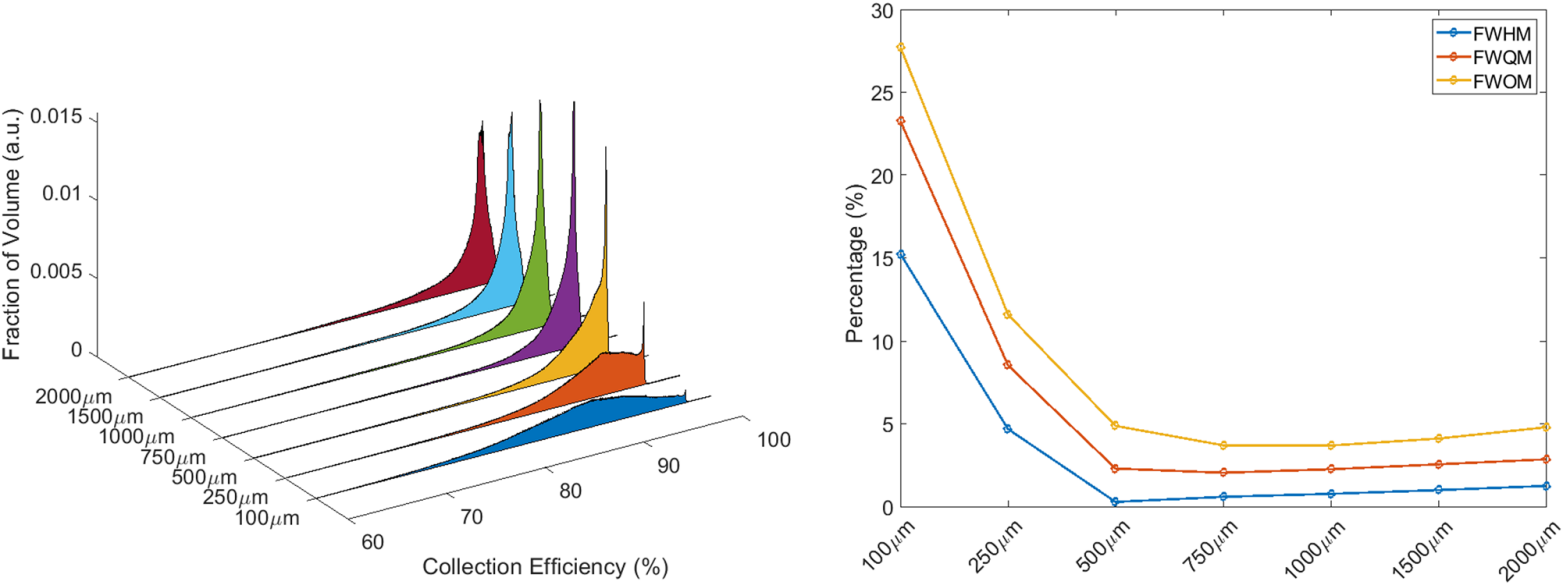
PAs plot of all simulated detectors as a function of the pixel size (left) and dependence of FWHM, FWQM and FWOM to pixel dimensions (right). The minimum of FWQM is at 750 μm.

PAs of all simulated detectors are reported in Fig. 4. The pixel dimension which minimises FWQM and FWOM is 750 μm, while the 500 μm pixel diameter is slightly better when looking at FWHM. Anyway, since PWEF considers the peak asymmetry and its value is minimised by the 750 μm diameter, we consider that as the optimal pixel size identified by the simulations.

### Fabrication and characterization

Six crystals 10 × 10 × 5 mm^3^ were fabricated in quasi-hemispherical configuration with three different pixel diameters: 250 µm, 750 µm and 1500 µm. The intermediate value was selected as it is the optimal size according to the simulation results, while the other two values were chosen to verify that the minimum computationally found was correct. For each pixel dimension, two sensors were fabricated to take in account the variability that can arise during the realisation. The largest diameter allows also to test the advantage in the ease of the bonding step by evaluating the change of the final performances in comparison to the ideal pixel.

The first test to determine the sensor quality is the measurement of the dark leakage current, i.e., the current flowing inside the polarised detector in absence of radiation. The operating condition (bias) was set considering the following trade-off: higher electric fields speed up the charge extraction and reduce carrier trapping; lower electric fields lead to less leakage current that otherwise would worsen the signal-to-noise ratio.

**Figure 5:**
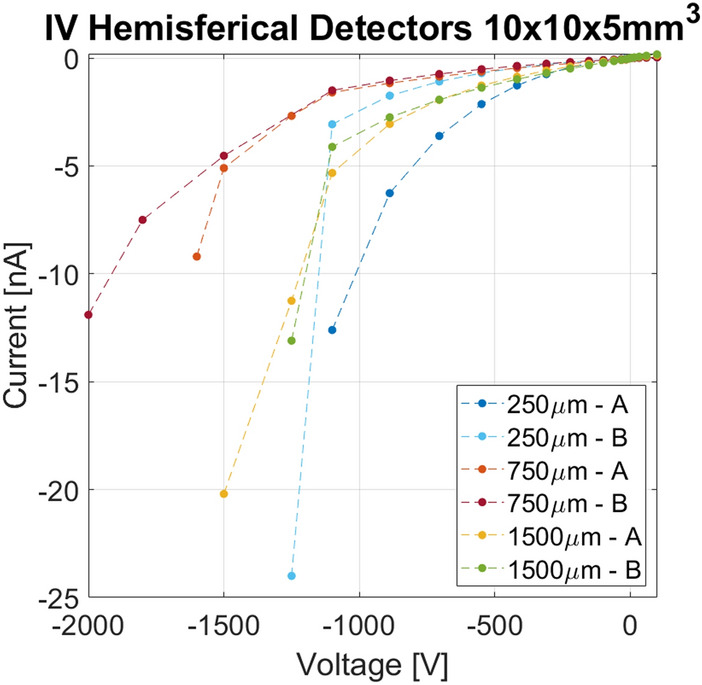
Current–voltage curves for the six fabricated detectors. Absolute current values increase more rapidly for the sensor that was damaged during the process. Measurements were carried out at 20 °C.

The two detectors of each pair with identical pixel size are indicated with the letters "A" and "B". The I–V curves are shown in Fig. 5 and appear to be reproducible for the couples with the same pixel dimension. Absolute current values never exceed 25 nA at 1250 V or higher voltages for the best sensors. This is a necessary condition to ensure good spectroscopic performances in the low energy range. Low surface leakage current also ensures that the simulated electric field is not affected by distortion due to surface effects.

**Figure 6:**
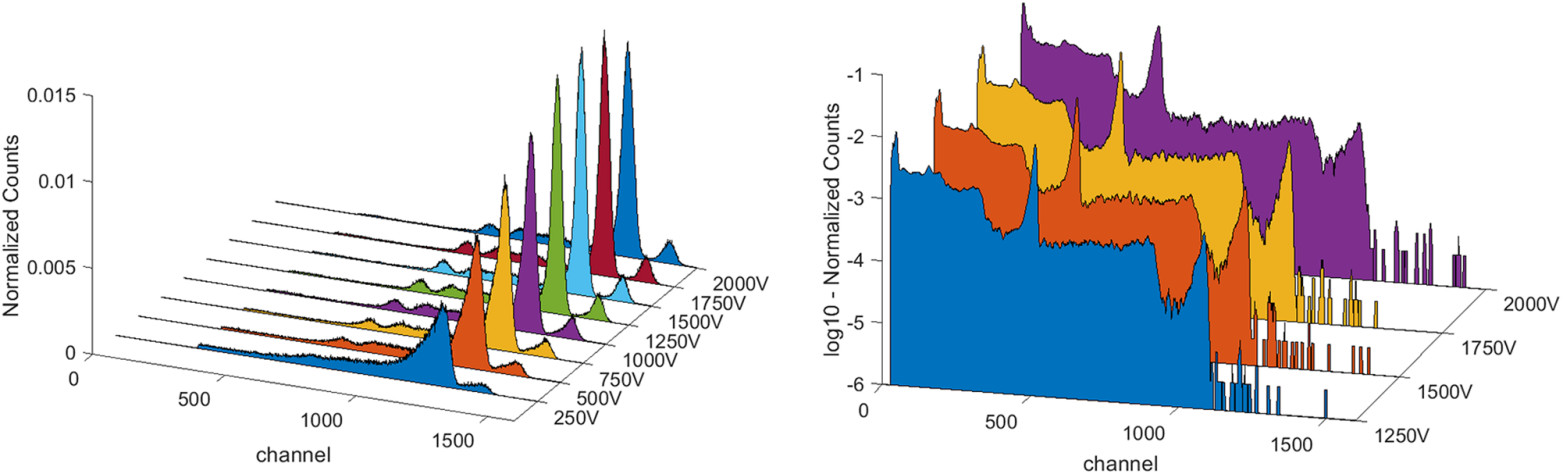
Co-57 (left) and Na-22 (right) spectra acquired with detector V5 750A at different bias voltages. Measurements were carried out at 20 °C.

To determine the voltage that maximises charge collection, several spectra were acquired increasing the bias voltage from 250 to 2000 V, with a step of 250 V. The voltage range for this test was chosen taking care that dark leakage current remained below 20 nA. In this way the minimum energy resolution of the main photopeak in the spectrum was found, when the tail effect due to hole trapping is limited. The optimal polarisation voltage was initially evaluated with Co-57 source (Fig. 6-left), whose radiation is absorbed within the first few millimetres of CZT, and then with Na-22 (Fig. 6-right) that has a more energetic emission and it irradiates the whole sensor volume. FWHM values relative to the radioisotopes photopeaks are reported in Table 2 with the corresponding voltages.

**Table 2:** Energy resolution obtained at 122 and 511 keV for the six detectors. The detector name refers to the anode diameter in µm. The bias voltage indicated is the one related to the best FWHM obtained. Measurements were carried out at 20 °C.

Detector	Bias voltage (V)	FWHM Co-57 (%) (122 keV)	Bias voltage (V)	FWHM Na-22 (%) (511 keV)
1500A	1250	3.3	1500	1.7
1500B	750	3.2	1250	1.8
750A	1250	2.8	1500	1.4
750B	1500	3.1	1500	1.8
250A	1000	4	1000	2.8
250B	1000	4.2	1250	2.2

**Figure 7:**
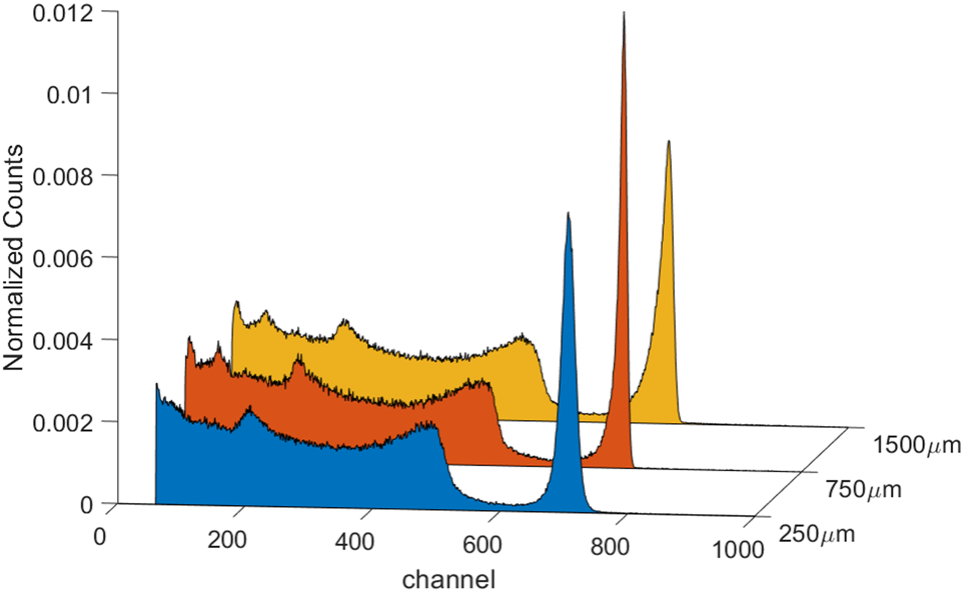
Detector performance comparison for the devices with the better resolution at their optimal working bias obtained measuring Cs-137. Detectors with 250 μm, 750 μm and 1500 μm pixels were compared. Measurements were carried out at 20 °C.

**Figure 8:**
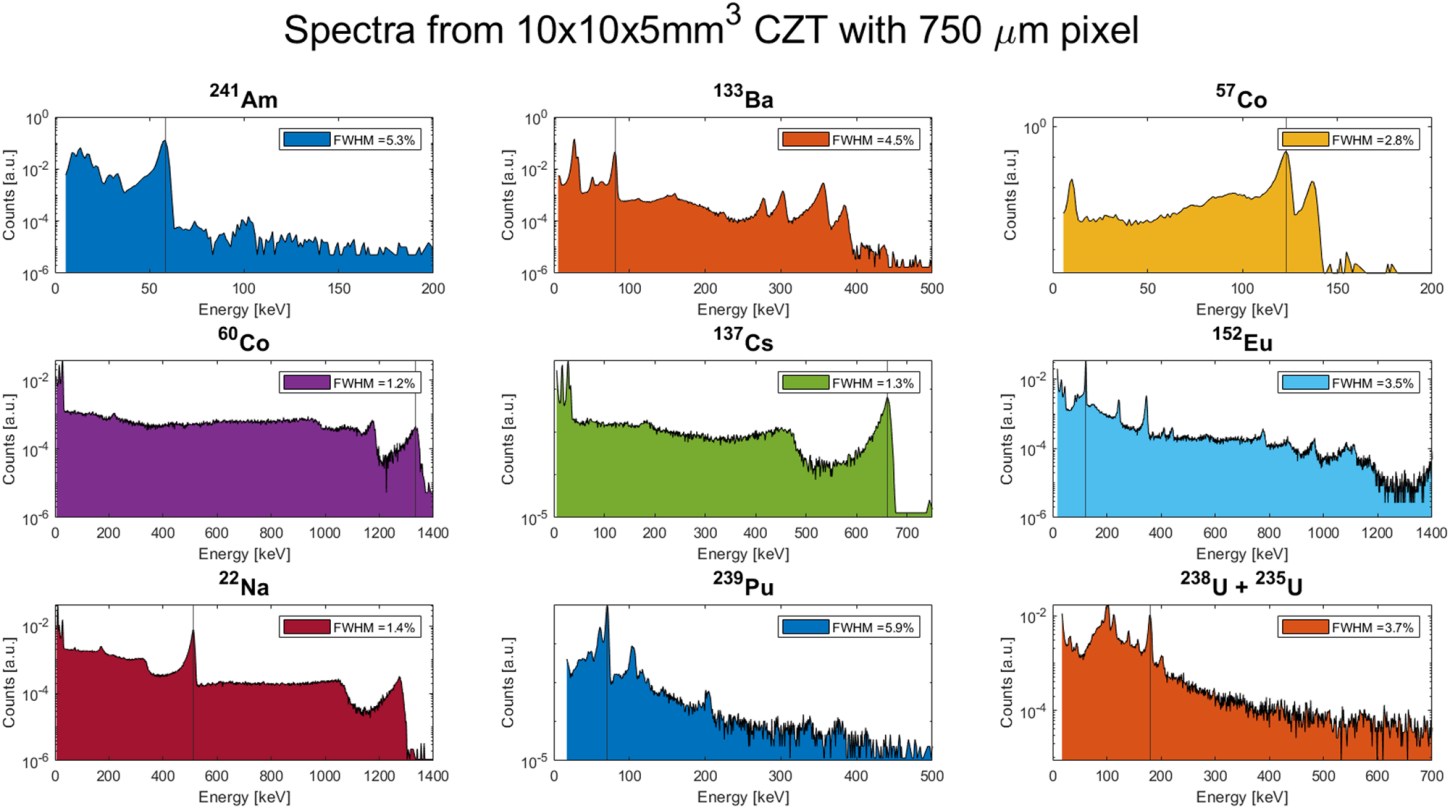
Spectra acquired with the best detector with a pixel of 750 µm, using nine different nuclear sources. The legend in the subplots reports the FWHM of the photopeak indicated by the vertical line. Measurements were carried out at 20 °C.

The best detector of each pair was selected for characterization with the Cs-137 source. Table 3 shows the results of these further measurements. As predicted by the simulations, the sensor with the best energy resolution was one of the pairs with 750 μm diameter, in particular the sensor 750A. An additional spectrum of the Co-60 source was also acquired in this case. In Fig. 7 the comparison among V5 detectors with different pixel sizes are represented. All the spectra acquired with the best 750 µm detector are shown in Fig. 8.

**Table 3:** FWHM of spectra from the reported sources acquired with the most performant detectors for each pixel dimension. The voltage in brackets is referred to a measure with a bias different from the one in the corresponding column. Measurements were carried out at 20 °C.

Detector	Voltage (V)	FWHM (%)			
		Co-57 (122 keV)	Na-22 (511 keV)	Cs-137 (662 keV)	Co-60 (1332 keV)
1500A	1500	3.4	1.7	1.7	–
750A	1500	4	1.4	1.3	1.2
250B	1250	4.2 (1000 V)	2.2	1.9	–

Measurements reflect the expected trend showing the best values for the 750 µm pixel anode; values are slightly worse increasing the pixel diameter to 1500 µm; the worst detector is the one with 250 µm pixel. These results are in perfect agreement with simulations.

Subsequently, a new batch of five V5 detectors was realised to verify the reproducibility of the obtained results. The performances of these detectors are described in the Supplementary information file.

### Large volume QHDs optimization

Once the simulator reliability was verified, detectors with a larger active volume were modelled and fabricated accordingly. 15 × 15 × 10 mm^3^ (V22) and 20 × 20 × 10 mm^3^ (V40) detectors crystals provided by Redlen were re-fabricated for this scope. Detectors with different pixel sizes were simulated using the same material parameters adopted for V5 detectors, except for the bias voltage that was set to 2000 V for both V22 and V40. Since the reliability of the simulator was already demonstrated, only detectors with the best simulated performance were fabricated and characterised.

### Simulations

The same approach adopted for V5 detectors was used for the simulations of V22 and V40 detectors. Several pixel dimensions ranging from 500 μm up to 5 mm were chosen, and relative space charge density distribution, electric field and weighting fields were simulated.

Simulation results show that V40 detectors behave similarly to V5 detectors, while the electric field profile of V22 detectors is different because their shape is L × L × (2/3)L and not L × L × (1/2)L. Indeed, the sensor aspect ratio strongly influences the value of the optimal pixel dimension.

**Figure 9:**
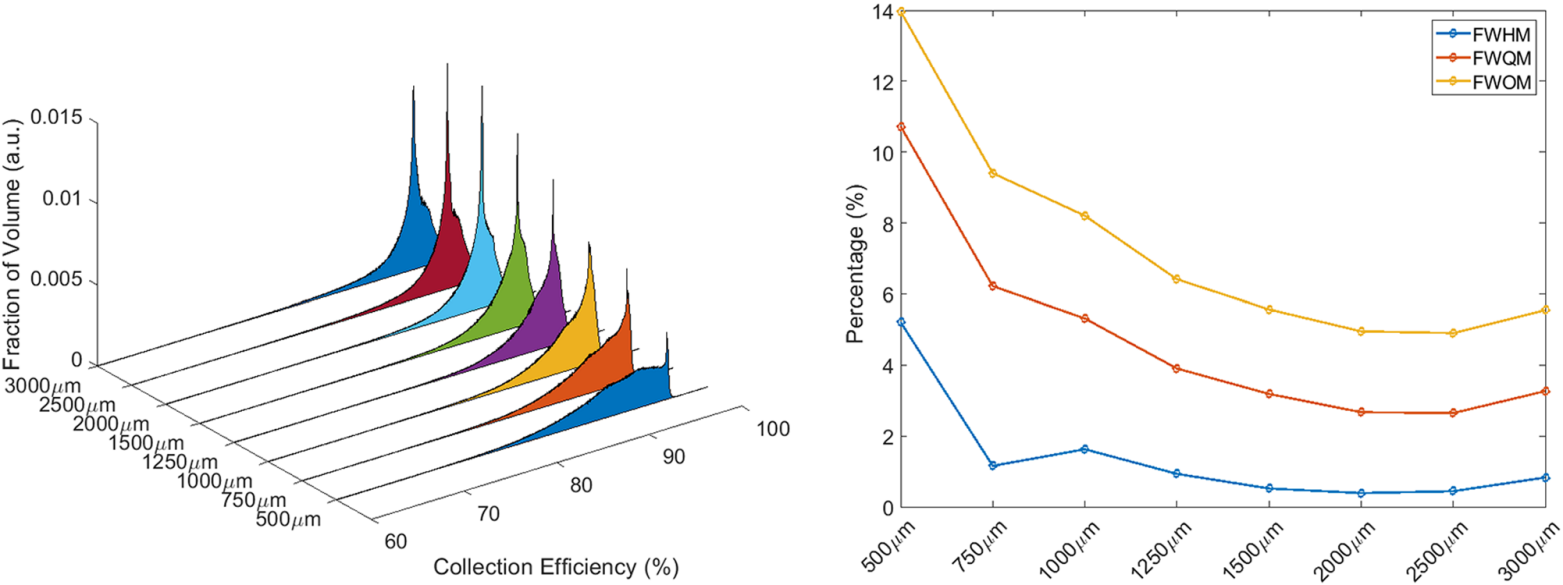
15 × 15 × 10 mm^3^ PAs obtained from detectors with different pixel sizes (left). Dependence of FWHM, FWQM, and FWOM on pixel size (right).

**Figure 10:**
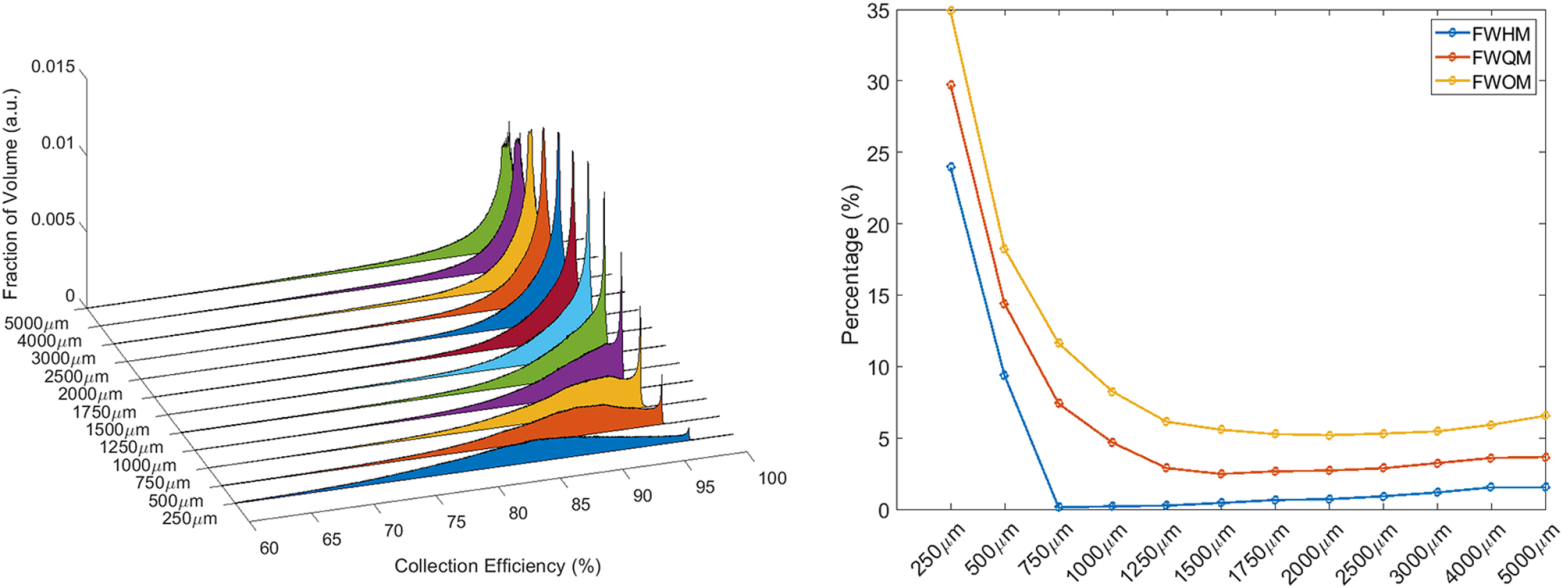
20 × 20 × 10 mm^3^ PAs obtained from detectors with different pixel sizes (left). Dependence of FWHM, FWQM, and FWOM on pixel size (right).

PAs were evaluated for V22 (Fig. 9—left) and V40 detectors (Fig. 10—left) and relative FWHM, FWQM and FWOM are shown in Figs. 9 and 10 (right). Thanks to the PWEF it was possible to identify the best pixel dimensions for both detector types: 2 mm and 1.25 mm are the optimal pixel dimensions respectively for V22 and V40 detectors.

It must be noted that the V22 sensor shows a best pixel size much larger than the other simulated geometries (V5 and V40), which is reasonably due to the different form factor of the detector.

### Fabrication and characterization

According to results suggested by simulations, two 15 × 15 × 10 mm^3^ detectors with a pixel of 2 mm and four 20 × 20 × 10 mm^3^ detectors with a pixel of 1.25 mm were realised. V22 and V40 were prepared and bonded to dedicated 3D printed sample holders following the same procedure described for V5 detectors. The four V40 detectors and the two V22 detectors were fabricated in the same batch and any remarkable issue with surface preparation, electrode deposition or surface passivation occurred, despite the handling of such large volume crystals was challenging.

**Figure 11:**
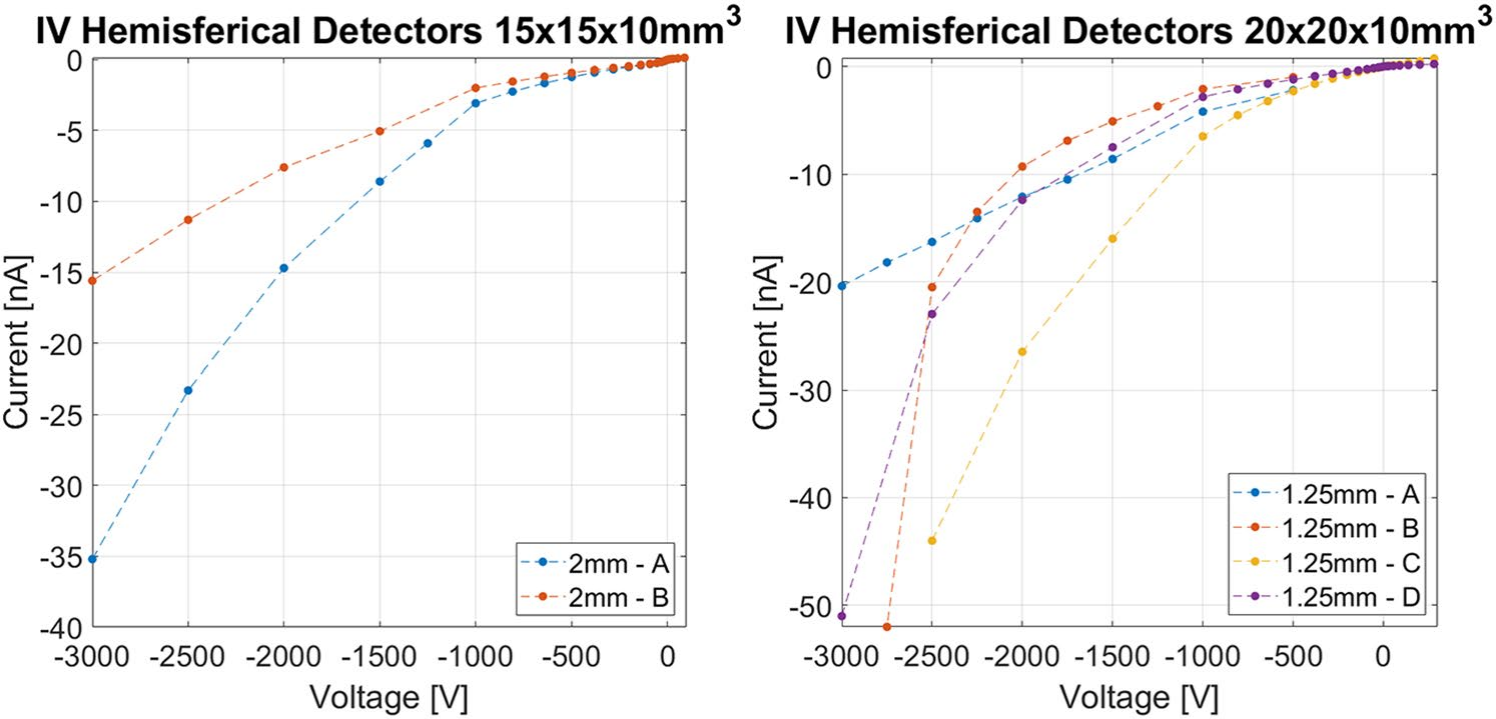
Current–voltage curves for the 15 × 15 × 10 mm^3^ hemispherical detectors (left) and for the 20 × 20 × 10 mm^3^ ones (right). Measurements were carried out at 20 °C.

The current–voltage curves of V22 and V40 detectors are reported in Fig. 11. I–V curves allowed us to choose the optimal voltage bias range of the detectors.

Both V22 detectors worked properly and similarly, so that only the results related to 2000B (2 mm—B) are shown. Three of the four V40 detectors showed poor spectroscopic properties in spite of the reasonable leakage current, thus only the spectroscopic performances of sensor 1250A are shown. Since they were fabricated in the same batch, we suppose that these differences among them are due to CZT material inhomogeneity that affects the detector response, particularly when large detectors are realised.

**Figure 12:**
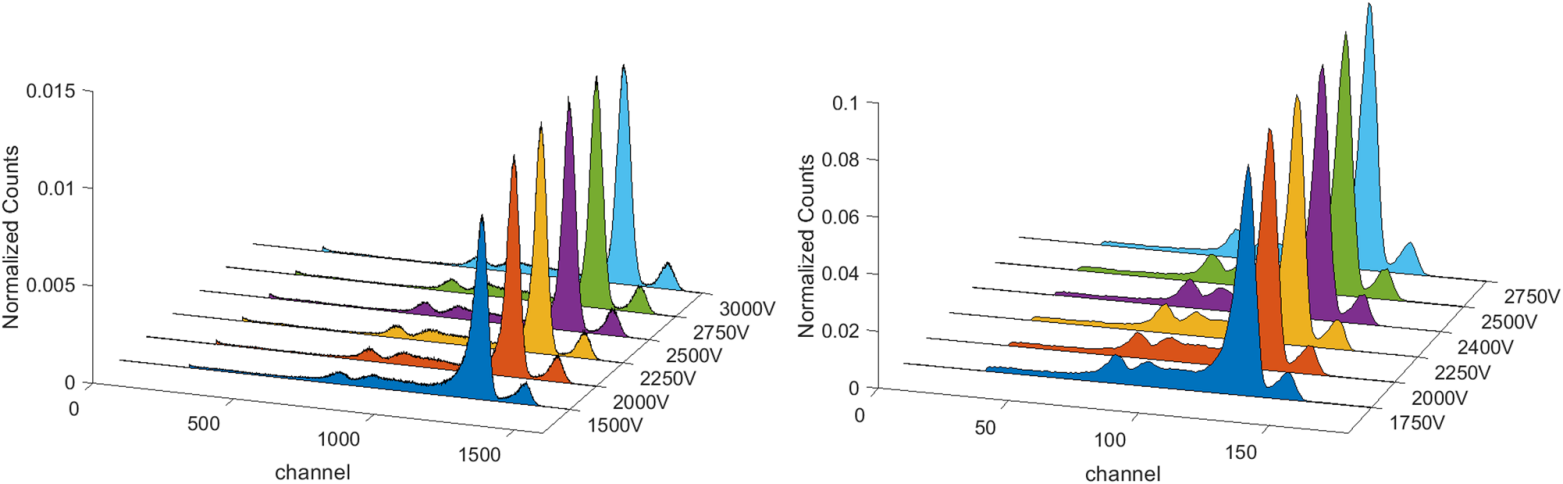
Co-57 spectra acquired from detector V22 2000B (right) and from detector V40 1250A (left) with different bias voltages. Measurements were carried out at 20 °C.

Figure 12 shows the spectra of Co-57 radioactive source at different bias voltages for V22 2000B and V40 1250A. The optimal working bias voltage was 2250 V for V22 2000B and 2500 V for V40 1250A, selected by analysing the minimum FWHM of the main photopeak in these spectra, which is respectively equal to 3.8% and 4.5%.

The spectra of Na-22, Cs-137 and Co-60 source spectra were additionally acquired, polarising the sensors at a bias that optimise performances at 511 keV, as reported in Table 4. Table 4 also shows the FWHMs values experimentally measured.

**Table 4:** FWHM of spectra from the reported sources acquired with the most performant detector of type V22 and V40. The voltages in brackets are referred to measures with a bias different from the one in the corresponding row. Measurements were carried out at 20 °C.

Detector	Voltage (V)	FWHM (%)			
		Co-57 (122 keV)	Na-22 (511 keV)	Cs-137 (662 keV)	Co-60 (1332 keV)
V22 2000B	2750	3.8 (2250 V)	1.8	1.7	1.6
V40 1250A	3000	4.5 (2500 V)	2.9	2.7	3.2

Considering that a large volume of active material is read by a single electrode, V22 detectors have demonstrated excellent performances with an energy resolution below 2%. The V40 sensor that properly work showed an energy resolution lower than 3% for Cs-137 source.

**Figure 13:**
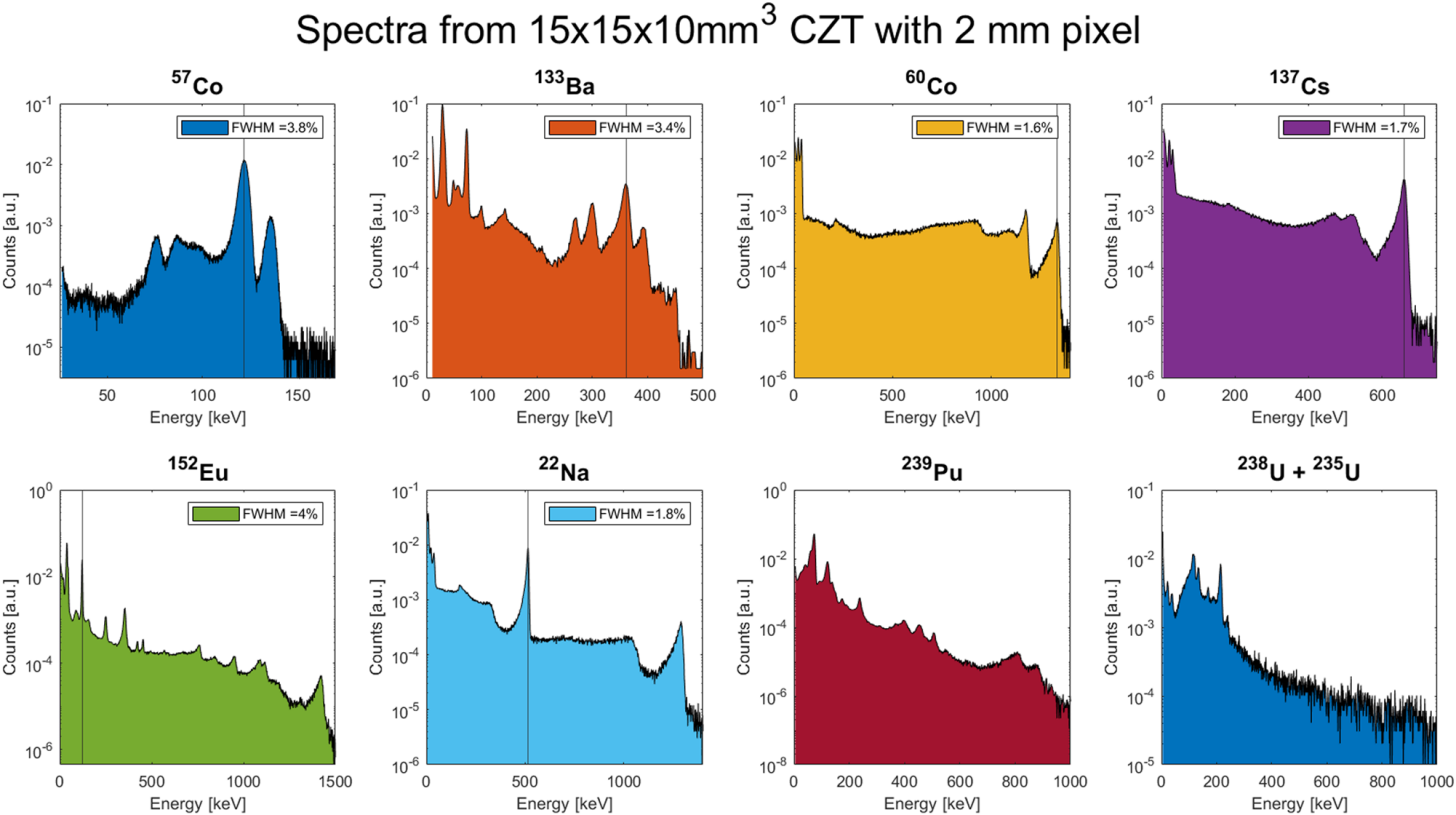
V22 2000B detector response to several nuclear sources. The legend in the subplots reports the FWHM of the photopeak indicated by the vertical line. Measurements were carried out at 20 °C.

**Figure 14:**
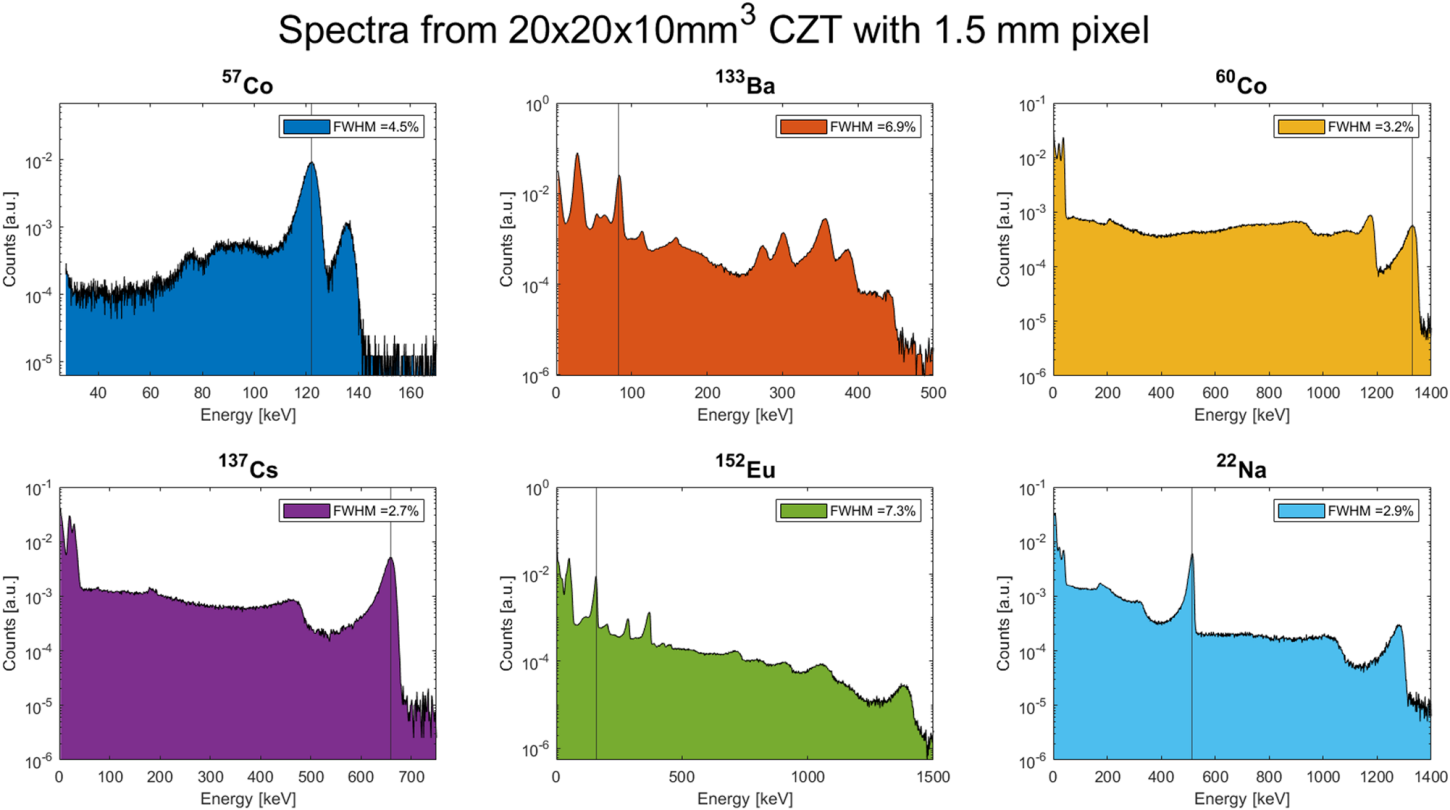
V40 1250A detector response to several nuclear sources. The legend in the subplots reports the FWHM of the photopeak indicated by the vertical line. Measurements were carried out at 20 °C.

The response of V22 2000B and V40 1250A detectors have also been characterised using other nuclear sources such as Ba-133, Eu-152, P-239, and Uranium. All these spectra are shown in Figs. 13 and 14.

## Discussion

10 × 10 × 5 mm^3^ quasi-hemispherical detectors were simulated in order to identify the optimal pixel dimension. Six 10 × 10 × 5 mm^3^ detectors with three different pixel sizes were fabricated in order to validate the simulations. Experimental detector performances were in full agreement with simulator outcomes. Both simulation and experiments showed that the optimal pixel diameter for 10 × 10 × 5 mm^3^ QHDs is 750 μm. Reproducibility of the results was also verified by redundant fabrication of the same optimised geometry on different CZT crystals.

The importance of considering space charge distribution inside the QHDs in simulations was presented and demonstrated in this work. Simulations not including space charge distribution show an electric field distribution largely inhomogeneous and with a low electric field in most part of the detector volume, a feature that is not verified by experiments in real devices.

Bao et al.^15^ found a much larger optimal pixel size for 10 × 10 × 5 mm^3^ detectors: in their evaluation the optimal pixel diameter was 2.5 mm. However their simulation did not consider the space charge present inside the detector that was proven to be effective in this work. Moreover the results of simulations were not supported by experimental validation. On the contrary, simulations presented in this work included the effect of space charge and were supported by the experimental results obtained by the characterization of detectors with different pixel diameters.

The most performing detectors were used to acquire spectra of several nuclear sources. Considering that no pulse shape analysis was applied, noticeable spectroscopic resolution values were achieved by using digital readout electronics (FWHM 1.3% at 662 keV and 3.1% at 122 keV).

Once the simulator was validated, it was used to optimise the geometry of detectors with larger volumes. 15 × 15 × 10 mm^3^ and 20 × 20 × 10 mm^3^ detectors were modelled to identify the best pixel sizes that were found to be, respectively, 2 mm and 1.25 mm. Large volume QHDs were fabricated according to the simulations, and were subsequently electrically and spectroscopically characterised.

Even though 15 × 15 × 10 mm^3^ detectors are not characterised by the typical L × L × (½)L aspect ratio, remarkable energy resolution has been achieved (1.7% at 662 keV), taking into account the relatively large volume of the detector (2.2 cm^3^). This is probably thanks to the optimised value of the pixel dimension (2 mm).

When larger detector volumes are considered, as in the case of 20 × 20 × 10 mm^3^ detectors, satisfying energy resolution could be achieved (2.7% at 662 keV), but only for one detector over four fabricated. We believe that CZT material inhomogeneity negatively affects the spectroscopic response of the detectors for such large volume detectors (4 cm^3^).

The results presented in this work demonstrate that quasi-hemispherical geometry allows the realisation of large CZT-based detectors having excellent spectral resolution, without the need of any correction and requiring only a single electronic readout channel. Also, the results show the powerful exploitation of proper simulation tools for the optimization of detector geometry.

## Supplementary Information

Supplementary Information.

## Data Availability

All data necessary to replicate the experiments are included in the paper. Datasets generated and acquired during the current study are available from the corresponding author on a reasonable request.
