# Supplementary material for: Optimization of quasi-hemispherical CdZnTe detectors by means of first principles simulation
Source: Sci Rep. 2023 Feb 24;13:3212. doi: 10.1038/s41598-023-30181-2 (PMC9958116; doi:10.1038/s41598-023-30181-2)
Supplement: Supplementary file 1 — Supplementary Information. [file 41598_2023_30181_MOESM1_ESM.docx]

A low performing readout electronic chain was used to evaluate the detectors performance. In order to better compare the obtained results, also V5 0750A and V5 750B were measured with the same electronics. The used electronics chain is entirely analogic, it is composed by Amptek A250 CSP, A275 shaper and a commercial multichannel analyser (MCA 8000D).

Performances at 122 keV of new realised detectors are shown in Table 1. Mean FWHM obtained for these seven detectors with the best pixel size is 3.54±0.2 %, this result is perfectly aligned with FWHM values of first two detectors. This result was obtained thanks to the high homogeneity of 10x10x5 mm^3^ CZT crystal and the reproducibility of fabrication procedure.

| Detector | FWHM Co-57 [%]  (122 keV) |
| --- | --- |
| 750 A | 3.3 |
| 750 B | 3.7 |
| 750 C | 3.8 |
| 750 D | 3.4 |
| 750 E | 3.7 |
| 750 F | 3.6 |
| 750 G | 3.3 |

Table 1. Energy resolution obtained at 122 for spectra from Co-57 for V5 750A and the five detectors of the second batch.
